# The Thioamides Methimazole and Thiourea Inhibit Growth of *M. avium* Subspecies *paratuberculosis* in Culture

**DOI:** 10.1371/journal.pone.0011099

**Published:** 2010-06-14

**Authors:** Robert J. Greenstein, Liya Su, Sheldon T. Brown

**Affiliations:** 1 Department of Surgery, James J. Peters VA Medical Center, Bronx, New York, United States of America; 2 Laboratory of Molecular Surgical Research, James J. Peters VA Medical Center, Bronx, New York, United States of America; 3 Department of Medicine, James J. Peters VA Medical Center, Bronx, New York, United States of America; 4 Mt. Sinai School of Medicine, New York, New York, United States of America; Universita di Sassari, Italy

## Abstract

**Background:**

Thyrotoxicosis is conceptualized as an “autoimmune” disease with no accepted infectious etiology. There are increasingly compelling data that another “autoimmune” affliction, Crohn disease, may be caused by *Mycobacterium avium* subspecies *paratuberculosis* (MAP). Like *M. tb*, MAP is systemic. We hypothesized that some cases of thyrotoxicosis may be initiated by a MAP infection. Because other thioamides treat tuberculosis, leprosy and *M. avium* complex, we hypothesized that a mode of action of some thioamide anti-thyrotoxicosis medications may include MAP growth inhibition.

**Methods:**

The effect of the thioamides, thiourea, methimazole and 6-propo-2-thiouracil (6-PTU) were studied in radiometric Bactec® culture, on ten strains of three mycobacterial species (six of MAP, two of *M. avium* and two of *M. tb*. complex). Data are presented as “cumulative growth index,” (cGI) or “percent decrease in cumulative GI” (%-ΔcGI).

**Principal Findings:**

Methimazole was the most effective thioamide at inhibiting MAP growth. At 128µg/ml: MAP UCF-4; 65%-ΔcGI & MAP ATCC 19698; 90%-ΔcGI. Thiourea inhibited MAP “Ben” maximally; 70%-ΔcGI. Neither methimazole nor thiourea inhibited *M. avium* or *M. tb*. at the doses tested. 6-PTU has no inhibition on any strain studied, although a structurally analogous control, 5-PTU, was the most inhibitory thioamide tested.

**Significance:**

We show inhibition of MAP growth by the thioamides, thiourea and methimazole in culture. These data are compatible with the hypothesis that these thioamides may have anti-prokaryotic in addition to their well-established eukaryotic actions in thyrotoxic individuals.

## Introduction

Prevailing medical dogma conceptualizes thyrotoxicosis as an “autoimmune” disease, with no universally accepted causative etiology. The mechanism of the thionamide class of anti-thyroid medications is accepted to be due to direct action on the hyperactive eukaryotic thyroid tissue. [1] There are suggestions that these autoimmune concepts should be readdressed. [2]

There is an intriguing, unexplained, association between thyroid hyperactivity and other “autoimmune” diseases, notably “inflammatory” bowel disease [3] including Crohn disease (CD) [4], [5] and ulcerative colitis (UC.) [6]

As with thyrotoxicosis, the etiology of CD and UC is (are) not known. *Mycobacterium avium* subspecies *paratuberculosis* (MAP), causes a chronic wasting diarrheal disease in cattle called Johne disease [7], that is evocative of CD. There are increasing direct [8] (& see [9] for review) and circumstantial data [10], [11] that MAP is zoonotic.[12] Intriguingly, the presence of MAP has been documented in a patient with thyrotoxicosis. [13]

It is of considerable interest that the thioamides ethionamide and prothionamide are used in the therapy of leprosy, tuberculosis and *M. avium* complex infections diseases[14], [15], and thiourea isoxyl is active against *M. tb*. [16] Additionally both of the antithyrotoxic medications Methimazole and Propothiouricil inhibit *M. leprae* in the mouse footpad model.[17]–[19]

Accordingly, we hypothesized that anti-thyrotoxicosis thioamide medications in addition to their multifold well documented eukaryotic actions [1], may have prokaryote activity in thyrotoxic individuals. Specifically, we hypothesized that these medications may interfere with the growth kinetics of MAP. The clinical responses to these anti-thyroid medications are idiosyncratic. Therefore, we further hypothesized that any MAP culture inhibition would be strain and agent specific.

We herein report on the effect on the growth kinetics on MAP, of the thioamide anti-thyroid medications methimazole and 6-propo-2-thiouricil (6-PTU) as well as thiourea, (an integral structural component of both methimazole and 6-PTU.) *M. leprae* cannot be grown in culture. [20] Therefore, as experimental control mycobacteria we studied the *M. avium* and the *M. tuberculosis* complexes.

## Methods

This study was approved by the Research & Development Committee at the VAMC Bronx NY (0720-06-038) and was conducted under the Institutional Radioactive Materials Permit (#31-00636-07).

### Bacterial Culture

Our Bactec® 460 (Becton-Dickinson Franklin Lakes NJ) ^14^C radiometric culture inhibition methods have previously been published in detail. [21]–[26] Because of interference with the assay [25], we do not use the detergent Tween 80 (recommended to prevent mycobacterial clumping) in culture. [27] Except for the amount of test agent, every vial has the identical concentration of all constituents (including identical 3.2% concentration of the dissolving agent, DMSO.) Vials are assayed on a daily basis, quantifying the amount of ^14^C released as ^14^CO_2_, by the integral detector in the Bactec 460. The data are obtained as a manufacturer determined, arbitrary Growth Units (GU) of 0-999.

In this study we evaluated ten strains of mycobacteria, six of which were MAP. Four MAP strains had been isolated from humans with Crohn's disease. “Dominic” (ATCC 43545), “Ben” (ATCC 43544) (both originally isolated by R. Chiodini [28]) and UCF 4 and ST-5 (both gifts of Saleh Naser, Burnett College of Biomedical Sciences, University of Central Florida, Orlando FL.)[8]. The other two MAP strains were from ruminants with Johne disease, ATCC 19698 and 303 (gift of Michael Collins Madison WI.) The *M. avium* subspecies *avium* strains (hereinafter called *M. avium*) were ATCC 25291 (veterinary source) and *M. avium* 101 [29]. To study the *M. tuberculosis* complex, we used two BioSafety level 2 strains, Bacillus Callmette Guerin (BCG) *M. bovis* Karlson & Lessel (ATCC 19015) and an avirulent *M. tb* strain; ATCC 25177 (all ATCC from ATCC Rockville MD).

The agents used to treat thyrotoxicosis that we studied were: 6-Propylthiouracil (6-propyl-2-thiouracil; 6-PTU; Sigma Cat # P3755) and methimazole (1-Methyl-2-imidazolethiol, 2-Mercapto-1-methylimidazole; Sigma Cat # M8506).[1], [30] We additionally studied Thiourea (Sigma Cat # T8656), an integral structural component found in both 5-PTU and methimazole (See Fig 1 in [1]). As an additional control, we studied a structural analog of PTU, 5-propyl-2-thiouracil (5-PTU; Sigma Cat # P0643.) Our positive antibiotic controls was monensin [24] and the negative control was the gluterimide antibiotic phthalimide.[25]

**Figure 1:**
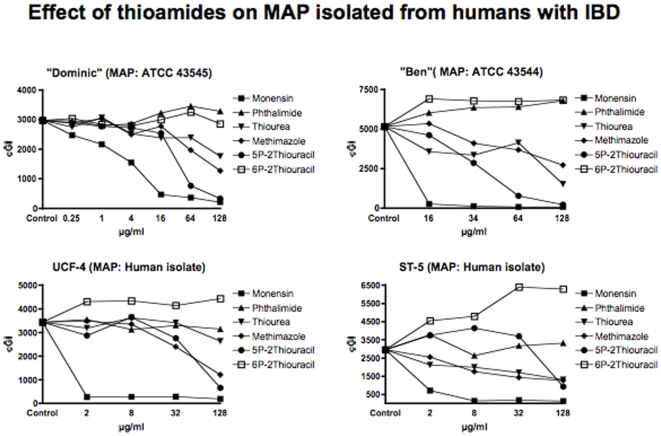
Shown are the cumulative Growth Indices (cGI) for the four MAP strains isolated from humans with Crohn disease. The positive control is Monensin, and the negative control is Phthalimide. Note that 5-PTU is consistently the most effective agent, and 6-PTU exhibits no inhibition. Methimazole is consistently more effective than Thiourea.

Chemical were dissolved in DMSO, aliquoted, stored at −80°C, thawed, used once and discarded. (All Sigma, St Louis. MO.) Agents were studied at concentrations ranging from 0.25 to 128 µg/ml (See Figures.)

For clarity and ease of understanding data are presented in two ways. Graphically (Figures 1–4) we present data for individual mycobacteria from a single experiment. Data are presented as the cumulative Growth Index (cGI.) The same data are then manipulated mathematically (1) and are presented (see Tables 1–6) as change in growth kinetics as the “percent change from control cGI” (Increase as “%+ΔcGI” or Inhibition; “%−ΔcGI”) Each Table has data from a single chemical agent but every mycobacterium studied. This is in contrast to the Figures where each graph is for an individual mycobacterium.

**Figure 2 pone-0011099-g002:**
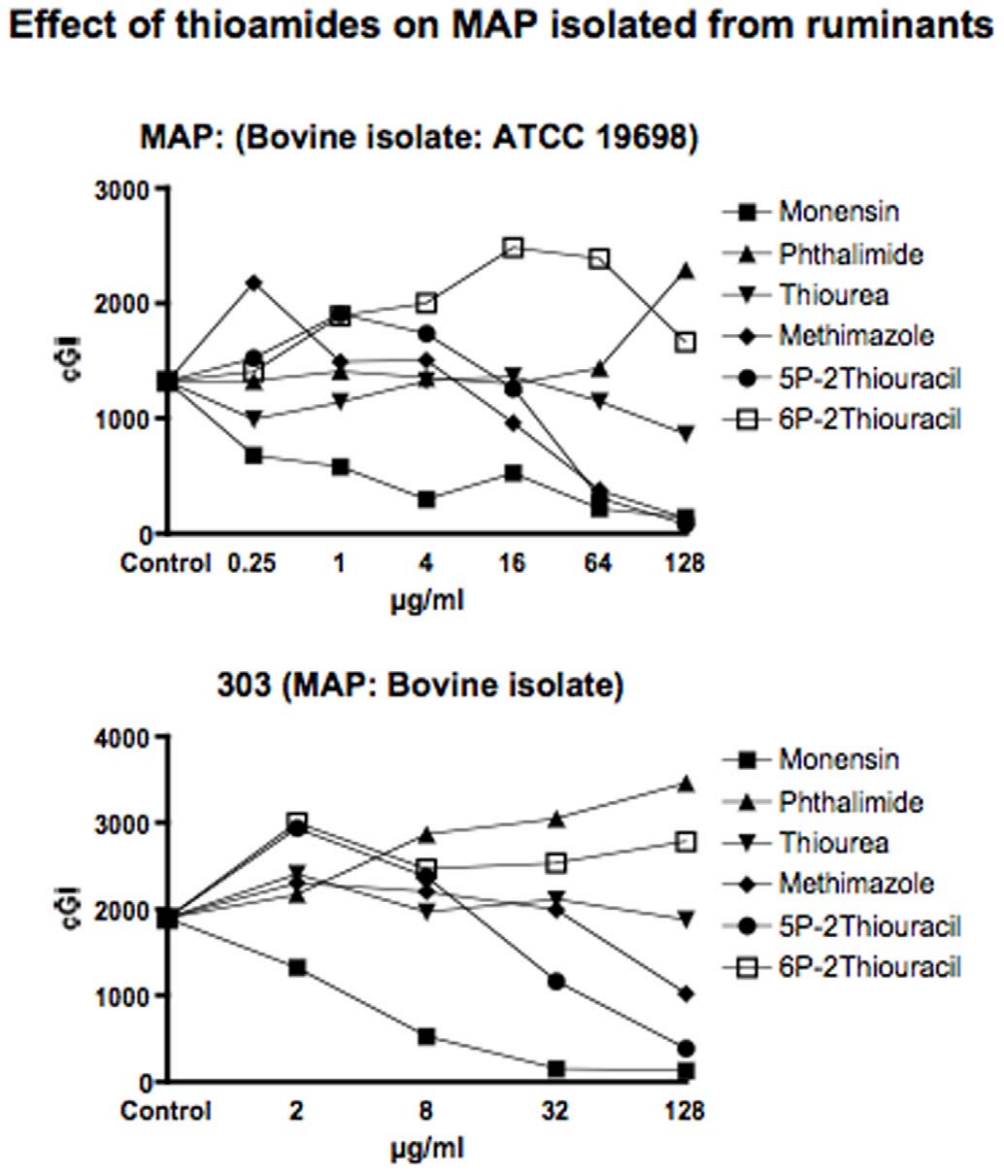
Shown are the cumulative Growth Indices (cGI) for the two MAP strains isolated from ruminants with Johne disease. The positive control is Monensin, and the negative control is Phthalimide. Note that 5-PTU is consistently the most effective agent, and 6-PTU exhibits no inhibition. Methimazole inhibits growth. Thiourea does not inhibit MAP 303.

**Figure 3 pone-0011099-g003:**
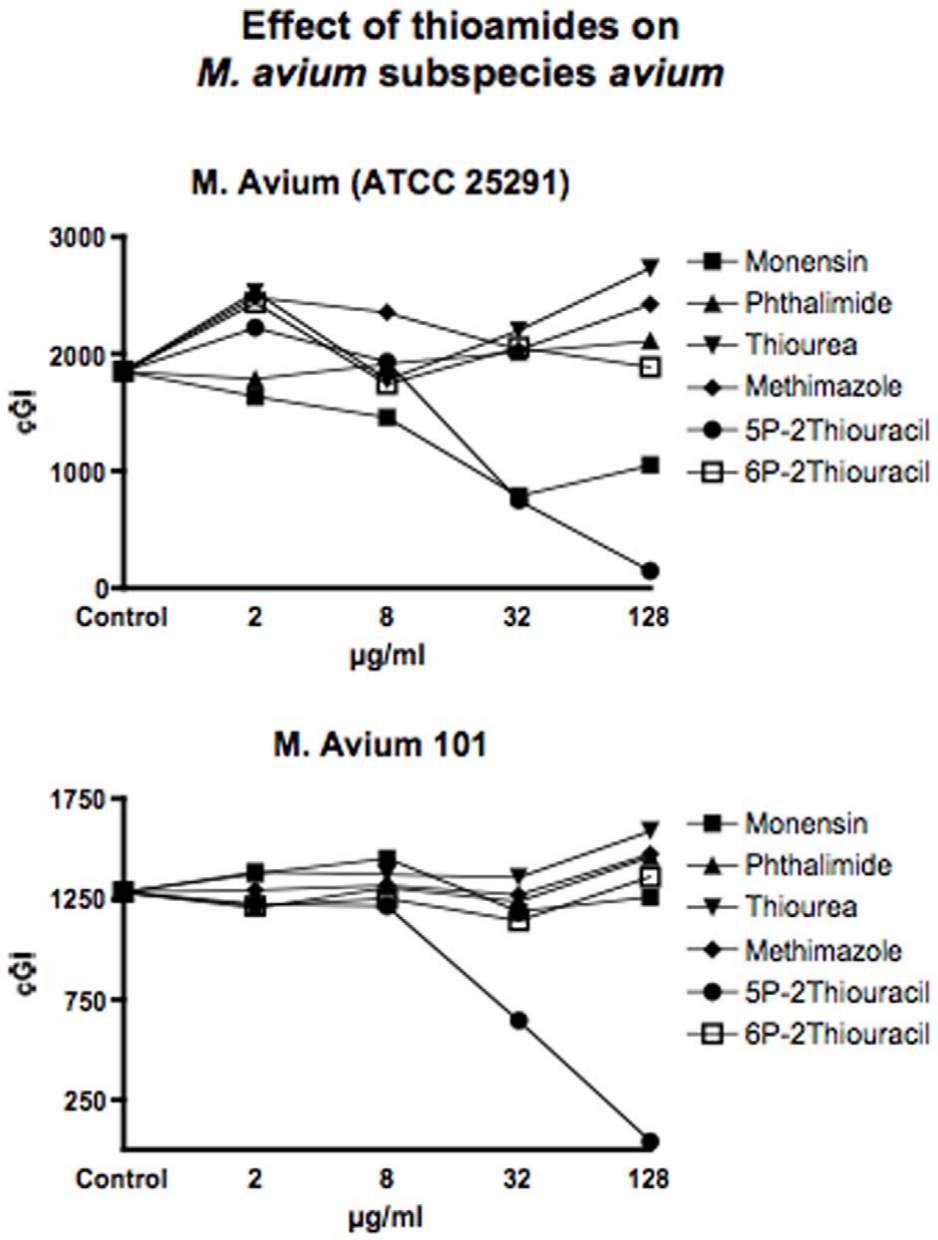
Shown are the cumulative Growth Indices (cGI) for the two *M. avium* strains. The positive control is Monensin, and the negative control is Phthalimide. Note that 5-PTU is consistently the most effective agent. Monensin does not inhibit *M. avium* 101, replicating previous findings. [24]

**Figure 4:**
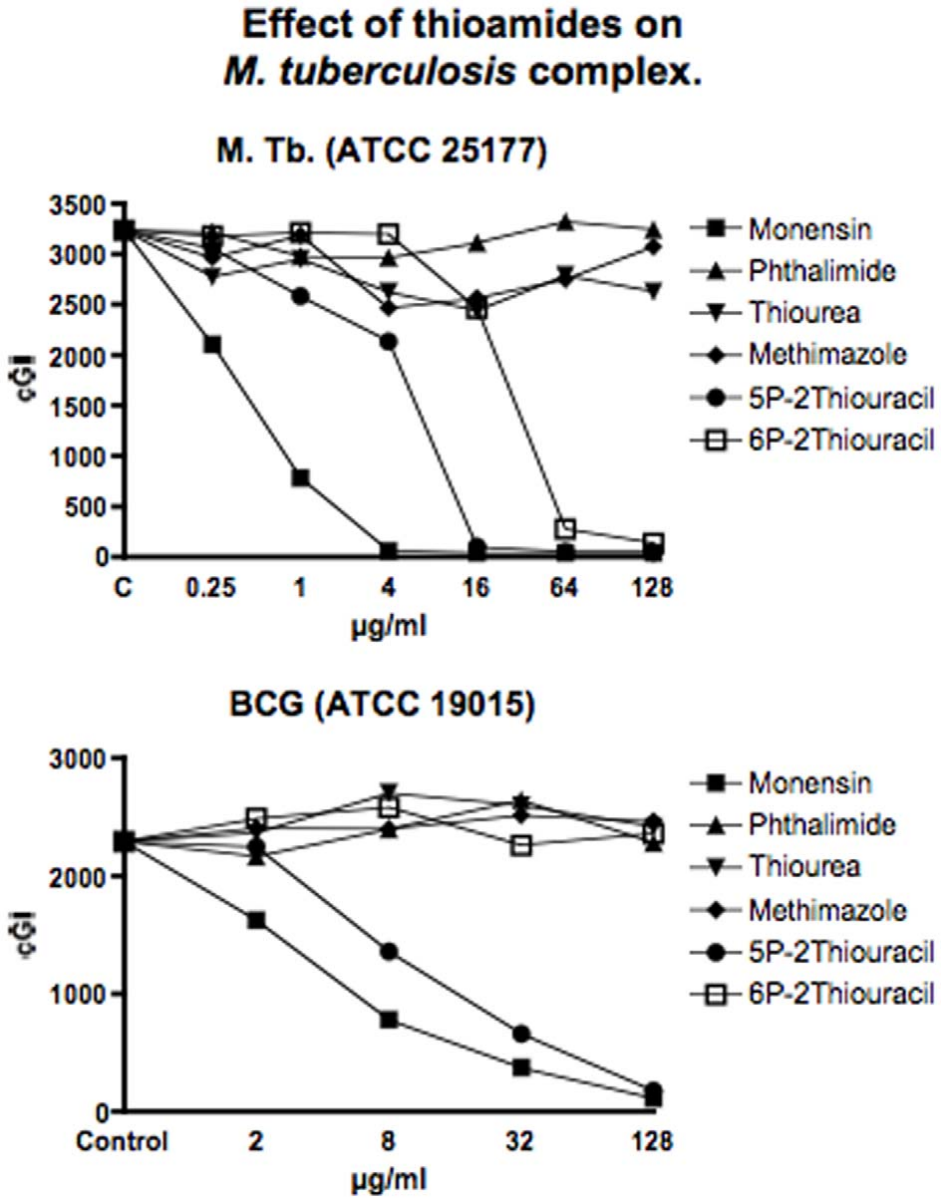
Shown are the cumulative Growth Indices (cGI) for the two *M. tb* complex strains. The positive control is Monensin, and the negative control is Phthalimide. 5-PTU is consistently the most effective agent. Note that, uniquely in this study, 6-PTU inhibits the BioSafety level 2 avirulent strain of *M. tb* (ATCC 25177) that we study.

**Table 1:** Positive Control: Monensin.

Dose Range µg/ml	MAP						M. avium		M. tb. complex	
	Human				Bovine				M. tb	BCG
	Dominic	UCF4	Ben	ST-5	19698	303	25291	101	25177	19015
Minimal	−27%	−92%	−95%	−76%	−56%	−30%	−12%	7%	−76%	−29%
Low	−48%	−92%	−98%	−95%	−78%	−72%	−21%	13%	−98%	−66%
Medium	−84%	−92%	−98%	−94%	−60%	−92%	−58%	−8%	−99%	−84%
Maximal	−91%	−94%	−99%	−95%	−90%	−93%	−43%	−2%	−98%	−95%

Our positive control antibiotic causes dose dependent inhibition in all but *M. avium* 101.

Shown are the effect of the test agent as either enhancement (%+ΔcGI) or inhibition of growth (-  =  %−ΔcGI) compared to control vials containing the same concentration of DMSO. See Methods for calculating %−ΔcGI. The dose ranges are “Minimal”  = 0.25–2 µg/ml: “Low”  = 4–8 µg/ml: “Medium”  = 16–32 µg/ml and “Maximal”  = 64–128 µg/ml. In these Tables “Maximal” is always the 128µg/ml data. For actual dose tested in individual experiments see the Abscissa on individual Figures.

**Table 2 pone-0011099-t002:** Negative Control: Phthalimide.

Dose Range µg/ml	MAP						M. avium		M. tb. complex	
	Human				Bovine				M. tb	BCG
	Dominic	UCF4	Ben	ST-5	19698	303	25291	101	25177	19015
Minimal	−6%	3%	17%	28%	7%	15%	−3%	−6%	−8%	−6%
Low	−4%	−9%	23%	−11%	2%	51%	3%	2%	−8%	5%
Medium	9%	−4%	24%	7%	−2%	61%	9%	−4%	−4%	15%
Maximal	11%	−9%	32%	12%	73%	83%	14%	14%	0%	−1%

Our negative control antibiotic has no inhibition in any of the ten mycobacterial strains tested. See Legend to Table 1 for explanation.

**Table 3 pone-0011099-t003:** Thiourea.

Dose Range µg/ml	MAP						M. avium		M. tb. complex	
	Human				Bovine				M. tb	BCG
	Dominic	UCF4	Ben	ST-5	19698	303	25291	101	25177	19015
Minimal	3%	−7%	−31%	−28%	−13%	27%	37%	8%	−9%	3%
Low	−15%	5%	−35%	−33%	1%	4%	−4%	7%	−19%	18%
Medium	−20%	−1%	−20%	−43%	4%	12%	19%	6%	−25%	13%
Maximal	−41%	−23%	−70%	−56%	−35%	−1%	48%	24%	−19%	5%

The basic thioamide molecule causes dose dependent inhibition four MAP strains isolated from humans but none of the other six strains. See Legend to Table 1 for explanation.

**Table 4 pone-0011099-t004:** Methimazole.

Dose Range µg/ml	MAP						M. avium		M. tb. complex	
	Human				Bovine				M. tb	BCG
	Dominic	UCF4	Ben	ST-5	19698	303	25291	101	25177	19015
Minimal	2%	1%	4%	−14%	13%	22%	34%	1%	−2%	5%
Low	−16%	−3%	−20%	−41%	14%	16%	27%	3%	−24%	5%
Medium	−7%	−30%	−28%	−52%	−27%	5%	10%	−1%	4%	10%
Maximal	−57%	−65%	−47%	−57%	−90%	−46%	31%	15%	−5%	7%

Note dose dependent inhibition in five of six MAP strains, but none of the four Control strains. See Legend to Table 1 for explanation.

**Table 5 pone-0011099-t005:** 5P-2Thiouracil.

Dose Range µg/ml	MAP						M. avium		M. tb. complex	
	Human				Bovine				M. tb	BCG
	Dominic	UCF4	Ben	ST-5	19698	303	25291	101	25177	19015
Minimal	−6%	−16%	−11%	27%	44%	55%	20%	−5%	−20%	−2%
Low	−8%	6%	−45%	40%	32%	25%	4%	−6%	−34%	−41%
Medium	−14%	−20%	−85%	25%	−5%	−39%	−60%	−50%	−97%	−71%
Maximal	−89%	−81%	−96%	−69%	−94%	−80%	−92%	−97%	−99%	−92%

With this structural analog of 6-PTU there is dose dependent inhibition in eight of ten strains and inhibition at 128 µg/ml in the remaining two. Note that *M. avium* 101 is inhibited. See Legend to Table 1 for explanation.

**Table 6:** 6P-2Thiouracil.

Dose Range µg/ml	MAP						M. avium		M. tb. complex	
	Human				Bovine				M. tb	BCG
	Dominic	UCF4	Ben	ST-5	19698	303	25291	101	25177	19015
Minimal	−4%	25%	34%	54%	43%	58%	32%	−5%	−1%	8%
Low	−6%	26%	32%	62%	81%	30%	−6%	−2%	−1%	13%
Medium	1%	21%	31%	116%	88%	34%	11%	−11%	−24%	−1%
Maximal	−4%	29%	33%	112%	26%	47%	2%	6%	−96%	3%

The medication used clinically to treat thyrotoxicosis causes no inhibition in any MAP strain. “Medium” for ATCC 19698 is 64 µg/ml as 32 µg/ml was contaminated. 6-PTU causes dose dependent inhibition in only *M. tb* ATCC 25177. See Legend to Table 1 for explanation.

During the course of these experiments, for technical reasons, the doses tested were progressively modified. There were four ranges. In every experiment the doses ranged from “Minimal” (0.25–2 µg/ml), to “Low” (4–8 µg/ml,), to “Medium” 16–32 µg/ml and “Maximal” doses (64–128 µg/ml.) See individual Figures for actual dosage tested in each separate experiment. See [22] for calculation of %−ΔcGI. In the data presented in the Tables, the “Maximal” dose is always the 128µg/ml value.

## Results

As previously [24], in this study we show that all MAP strains are inhibited by Monensin (Table 1 and Figures 1 & 2.) As previously [24], Monensin does not inhibit one of our two *M. avium* control strains (*M. avium* 101: Table 1 and Figure 3.) In our initial Monensin manuscript [24], we had only studied BCG from the *M. tb* complex. We now additionally study a Biosafety level II non-virulent strain of *M. tb*. ATCC 25177. Thus, we find that Monensin is even more inhibitory against *M. tb*. ATCC 25177 (−98%−ΔcGI @ 4µg/ml. Table 1 & Figure 4) than it is against BCG.

The negative control that we use is Phthalimide, a gluterimide antibiotic that has no mycobacterial inhibition.[25] In this study, as previously, Phthalimide has no dose dependent inhibition against any of the mycobacterial strains we study (Table 2 and Figures 1–4.)

Thiourea, an integral structural component of both 6-PTU and methimazole, causes dose dependent inhibition of all four MAP strains isolated from humans (Table 3 and Figure 1). In contrast, thiourea does not inhibit either the two bovine MAP isolates (Table 3 and Figure 2) or any of our four mycobacterial controls species *M. avium* (Table 3 & Figure 3) or *M. tb*. (Table 3 & Figure 4).

Methimazole, causes dose dependent inhibition of all MAP strains (Table 4 and Figures 1& 2.) It is most inhibitory on a bovine MAP isolate ATCC 19698 (90%−ΔcGI at 128µg/ml.) Methimazole has no inhibition on our mycobacterial controls, *M. avium* (Table 4 & Figure 3) or *M. tb* complex (Table 4 & Figure 4).

At the doses tested, the anti thyroid medication 6-PTU causes no inhibition of MAP growth against any strain tested (Table 5 & Figures 1–4). As a control we compared this to a structural analog, 5-PTU. To our surprise, the control, 5-PTU markedly inhibits every mycobacterial strain we studied. (Table 6 and Figures 1–4.)

## Discussion

Our data show that both thiourea and methimazole cause strain specific, dose dependent inhibition of MAP in radiometric culture. Thiourea is more active against the MAP strains isolated from humans and less active against MAP strains isolated from ruminants. Methimazole inhibits all MAP strains studied. In contrast, neither methimazole nor thiourea has any dose dependent inhibition against our *M. avium* and *M. tb*. control strains. These data could explain the lack of a consistent response to the medical therapy of clinical thyrotoxicosis.

Other thioamides have antimycobacterial activity in leprosy, tuberculosis and in *M. avium* complex infections. [14]–[16] Methimazole and 6-PTU inhibit *M. leprae*.[17]–[19] In our assay neither Methimazole nor 6-PTU inhibits *M. avium* subspecies *avium* or the *M. tb* complex. We conclude that the inhibition of growth by these antithyroid thioamides is specific to MAP and *M. leprae* [17]–[19], but not to mycobacteria in general.

Our data show no inhibition by 6-PTU on any of the ten mycobacterial strains we evaluated. These data have multiple possible explanations. 6-PTU is actively concentrated *in vivo* by both lymphocytes (by 666%) [31] and thyroid tissue. [32], [33] Thus the doses achieved *in vivo* may well exceed the concentrations used in our culture inhibition study.

*In vivo* there are multiple metabolites of 6-PTU. [34], [35] Some of these 6-PTU metabolites may have anti-MAP activity. This would be analogous to 5-ASA inhibiting MAP in culture whereas intact sulfasalazine, a parent molecule of 5-ASA, has no antiMAP activity in culture. [22] We have not been able to identify a commercial source of any 6-PTU metabolites [34], [35] to test in our culture inhibition system.

However, we were able to obtain 5-PTU a structural analog of 6-PTU. 5-PTU markedly inhibits all ten strains of mycobacteria that we studied. We are unaware of any studies that have evaluated the safety or efficacy of 5-PTU in the therapy of thyrotoxicosis. Nor does this study attempt to correlate the clinical doses given and tissue levels achieved *in vivo*, with antimycobacterial activity in tissue culture.

The time required to achieve a clinical response in the therapy of tuberculosis [36], leprosy [37] and IBD [38] is months. Likewise, a clinical response to thioamide anti-thyrotoxicosis medication requires months. [1], [2] This tardiness is ascribed to substantial reserves of thyroid hormone, which must be depleted before a clinical response can be observed. Our data are compatible with an alternative hypothesis. Mycobacteria replicate very slowly. We suggest that successful treatment of a mycobacterial trigger for thyrotoxicosis would take months to manifest clinically.

Other than a single report [13], to our knowledge an association between MAP and thyrotoxicosis has not been previously reported. This may be because detecting mycobacteria is not possible in some forms of mycobacterial diseases such as tuberculoid leprosy [39] or MAP in humans. [12] We suggest that to understand human MAP infections, more insights will be gained from analogies with leprosy [10], [11] than with tuberculosis.

The thioamides used to treat thyrotoxicosis have anti *M. leprae* effects in an animal model. [17]–[19] We conclude this prokaryotic inhibition may have therapeutic implications in thyrotoxicosis. Our data are compatible with the hypothesis that some cases of “autoimmune” thyroid disease may be instigated by a mycobacterial, specifically we posit a MAP, infection.

## References

[1] Cooper DS (2005). Antithyroid drugs.. N Engl J Med.

[2] Laurberg P (2006). Remission of Graves' disease during anti-thyroid drug therapy. Time to reconsider the mechanism?. Eur J Endocrinol.

[3] Bianchi GP, Marchesini G, Gueli C, Zoli M (1995). Thyroid involvement in patients with active inflammatory bowel diseases.. Ital J Gastroenterol.

[4] Inokuchi T, Moriwaki Y, Takahashi S, Tsutsumi Z, Ka T (2005). Autoimmune thyroid disease (Graves' disease and hashimoto's thyroiditis) in two patients with Crohn's disease: case reports and literature review.. Intern Med.

[5] Shah SA, Peppercorn MA, Pallotta JA (1998). Autoimmune (Hashimoto's) thyroiditis associated with Crohn's disease.. J Clin Gastroenterol.

[6] Jarnerot G, Azad Khan AK, Truelove SC (1975). The thyroid in ulcerative colitis and Crohn's disease. II. Thyroid enlargement and hyperthyroidism in ulcerative colitis.. Acta Med Scand.

[7] Johne HA, Frothingham L (1895). Ein eigenthumlicher fall von tuberculose beim rind (A particular case of tuberculosis in a cow).. Dtsch Zeitschr Tiermed, Vergl Pathol.

[8] Naser SA, Ghobrial G, Romero C, Valentine JF (2004). Culture of *Mycobacterium avium* subspecies *paratuberculosis* from the blood of patients with Crohn's disease.. Lancet.

[9] Greenstein R, Gillis T, Scollard D, Brown S, Fratamico P, Smith J, Brogden K (2009). Mycobacteria: Leprosy, a Battle Turned; Tuberculosis, a Battle Raging; Paratuberculosis, a Battle Ignored.. Sequelae and Long-Term Consequences of Infectious Diseases.

[10] Zhang FR, Huang W, Chen SM, Sun LD, Liu H (2009). Genomewide association study of leprosy.. N Engl J Med.

[11] Schurr E, Gros P (2009). A common genetic fingerprint in leprosy and Crohn's disease?. N Engl J Med.

[12] Greenstein RJ, Collins MT (2004). Emerging pathogens: is *Mycobacterium avium* subspecies *paratuberculosis* zoonotic?. Lancet.

[13] D'Amore M, Lisi S, Sisto M, Cucci L, Dow CT (2010). Molecular identification of Mycobacterium avium subspecies paratuberculosis in an Italian patient with Hashimoto's thyroiditis and Melkersson-Rosenthal syndrome.. J Med Microbiol.

[14] Wang F, Langley R, Gulten G, Dover LG, Besra GS (2007). Mechanism of thioamide drug action against tuberculosis and leprosy.. J Exp Med.

[15] Fajardo TT, Guinto RS, Cellona RV, Abalos RM, Dela Cruz EC (2006). A clinical trial of ethionamide and prothionamide for treatment of lepromatous leprosy.. Am J Trop Med Hyg.

[16] Phetsuksiri B, Jackson M, Scherman H, McNeil M, Besra GS (2003). Unique mechanism of action of the thiourea drug isoxyl on Mycobacterium tuberculosis.. J Biol Chem.

[17] Levy L, Anandan JA (1978). Further studies of the action of antithyroid drugs on Mycobacterium leprae.. Proc Soc Exp Biol Med.

[18] Levy L, Moon N (1972). Inhibition of the multiplication of Mycobacterium leprae by methimazole.. Am Rev Respir Dis.

[19] Levy L, Ullmann NM (1975). Inhibition of multiplication of Mycobacterium leprae by several antithyroid drugs.. Am Rev Respir Dis.

[20] Stewart-Tull DES, Ratledge C, Stanford J (1982). *Mycobacterium leprae* - The bacteriologist's enigma.. The Biology of the Mycobacteria, Volume 1: Physiology, Identification, and Classification.

[21] Greenstein RJ, Su L, Haroutunian V, Shahidi A, Brown ST (2007). On the Action of Methotrexate and 6-Mercaptopurine on M. avium Subspecies paratuberculosis.. PLoS ONE.

[22] Greenstein RJ, Su L, Shahidi A, Brown ST (2007). On the Action of 5-Amino-Salicylic Acid and Sulfapyridine on M. avium including Subspecies paratuberculosis.. PLoS ONE.

[23] Rastogi N, Goh KS, Labrousse V (1992). Activity of clarithromycin compared with those of other drugs against *Mycobacterium paratuberculosis* and further enhancement of its extracellular and intracellular activities by ethambutol.. AntimicrobAgents Chemother.

[24] Greenstein RJ, Su L, Whitlock R, Brown ST (2009). Monensin causes dose dependent inhibition of Mycobacterium avium subspecies paratuberculosis in radiometric culture.. Gut Pathogens.

[25] Greenstein RJ, Su L, Brown ST (2009). On the effect of thalidomide on *Mycobacterium avium* subspecies *paratuberculosis* in culture.. Int J Infect Dis.

[26] Greenstein RJ, Su L, Juste RA, Brown ST (2008). On the Action of Cyclosporine A, Rapamycin and Tacrolimus on M. avium including Subspecies paratuberculosis.. PLoS ONE.

[27] Damato JJ, Collins MT (1990). Growth of Mycobacterium paratuberculosis in radiometric, Middlebrook and egg-based media.. Vet Microbiol.

[28] Chiodini RJ, Van Kruiningin HJ, Thayer WJ, Coutu J (1986). Spheroplastic phase of mycobacteria isolated from patients with Crohn's disease.. J Clin Microbiol.

[29] Bertram MA, Inderlied CB, Yadegar S, Kolanoski P, Yamada JK (1986). Confirmation of the beige mouse model for study of disseminated infection with Mycobacterium avium complex.. J Infect Dis.

[30] Nakamura H, Noh JY, Itoh K, Fukata S, Miyauchi A (2007). Comparison of methimazole and propylthiouracil in patients with hyperthyroidism caused by Graves' disease.. J Clin Endocrinol Metab.

[31] Lam DC, Lindsay RH (1979). Accumulation of 2-[14C]propylthiouracil in human polymorphonuclear leukocytes.. Biochem Pharmacol.

[32] Marchant B, Alexander WD, Robertson JW, Lazarus JH (1971). Concentration of 35S-propylthiouracil by the thyroid gland and its relationship to anion trapping mechanism.. Metabolism.

[33] Marchant B, Alexander WD, Lazarus JH, Lees J, Clark DH (1972). The acclumulation of 35 S-antithyroid drugs by the thyroid gland.. J Clin Endocrinol Metab.

[34] Lindsay RH, Hulsey BS, Aboul-Enein HY (1975). Enzymatic S-methylation of 6-n-propyl-2-thiouracil and other antithyroid drugs.. Biochem Pharmacol.

[35] Lindsay RH, Kelly K, Hill JB (1979). Oxidative metabolites of [2-14C]propylthiouracil in rat thyroid.. Endocrinology.

[36] Small PM, Fujiwara PI (2001). Management of tuberculosis in the United States.. N Engl J Med.

[37] Britton WJ, Lockwood DN (2004). Leprosy.. Lancet.

[38] Podolsky DK (2002). Inflammatory bowel disease.. N Engl J Med.

[39] Ridley DS, Jopling WH (1962). A classification of leprosy for research purposes.. Lepr Rev.

